# Equitable suicide prevention for youth impacted by the juvenile legal system

**DOI:** 10.3389/fpsyt.2022.994514

**Published:** 2022-10-25

**Authors:** Jocelyn I. Meza, Sean Snyder, Caroline Shanholtz

**Affiliations:** ^1^Department of Psychiatry and Biobehavioral Sciences, David Geffen School of Medicine at University of California, Los Angeles, Los Angeles, CA, United States; ^2^Department of Psychiatry, Pennsylvania Hospital, Perelman School of Medicine, University of Pennsylvania, Philadelphia, PA, United States; ^3^Department of Psychology, University of California, Los Angeles, Los Angeles, CA, United States

**Keywords:** suicide, juvenile legal system, equity, adolescents, Sequential Intercept Model, suicide prevention and intervention, ethnoracially minoritized youth

## Abstract

Suicide is the second leading cause of death for adolescents in the United States. Despite the already alarmingly high rates of suicide attempts among adolescents, youth involved in the juvenile legal system (JLS) are up to three times more likely to have suicide attempts than their peers not impacted by the JLS. This public health crisis is also a matter of health equity, knowing that ethnoracially minoritized youth, mainly Black and Latinx youth, have disproportionate contact with the JLS. In order to disrupt the current elevated rates of suicide among Black and Latinx youth involved in the JLS, there needs to be more concerted efforts to improve assessment and suicide prevention efforts in the JLS. There are various potential touch points of care for suicide prevention and the Sequential Intercept Model (SIM), which outlines community-based responses to the involvement of people with mental and substance use disorders in the criminal justice system, can be used as a strategic planning tool to outline possible equitable interventions across these various touch points. Our purpose is to provide a comprehensive picture of gaps and equitable opportunities for suicide prevention across each intercept of the SIM. We provide recommendations of priorities to promote health equity in suicide prevention for ethnoracially minoritized youth impacted by the JLS.

## Introduction

Suicide is the second leading cause of death for adolescents in the U.S. (1). Historically, White youth have higher rates of death by suicide, when compared to Black and Latinx youth. However, these rates do not consider the rapid increase in suicide deaths and elevated rates of suicide attempts for Black and Latinx youth. Recent data suggests that Black and Latinx adolescent girls have the highest rates of suicide attempts (15.9 and 11.9%, respectively), compared to 9.4% of non-Hispanic White adolescent girls (2). When considering the different social systems in where Black and Latinx adolescents are disproportionately embedded in, youth in the juvenile legal system (JLS) are at even greater risk of suicide. Indeed, suicide rates are up to three times higher for youth impacted by the JLS than youth in the general population (3–5), and risk for suicide increases with greater involvement in the JLS (6).

The Sequential Intercept Model (SIM) was developed to describe various points in the criminal justice process at which individuals with mental illnesses could be diverted to alternative rehabilitative services and treatment (7). Heilbrun et al. (8) applied the SIM to the JLS and outline intercepts as (1) the first contact with either emergency services, (2) initial hearings and detention post-arrest, (3) jails and courts, (4) re-entry, and (5) community corrections. We expand this to include an intercept zero “Prevention,” separated court processes from confinement, and redefine confinement to include the juvenile-specific types of juvenile detention and long-term placements (either structured residential or psychiatric residential treatment centers). Our iteration of the SIM views the intercepts from a clinical perspective as various touchpoints for culturally responsive, trauma-informed suicide prevention.

**FIGURE 1:**
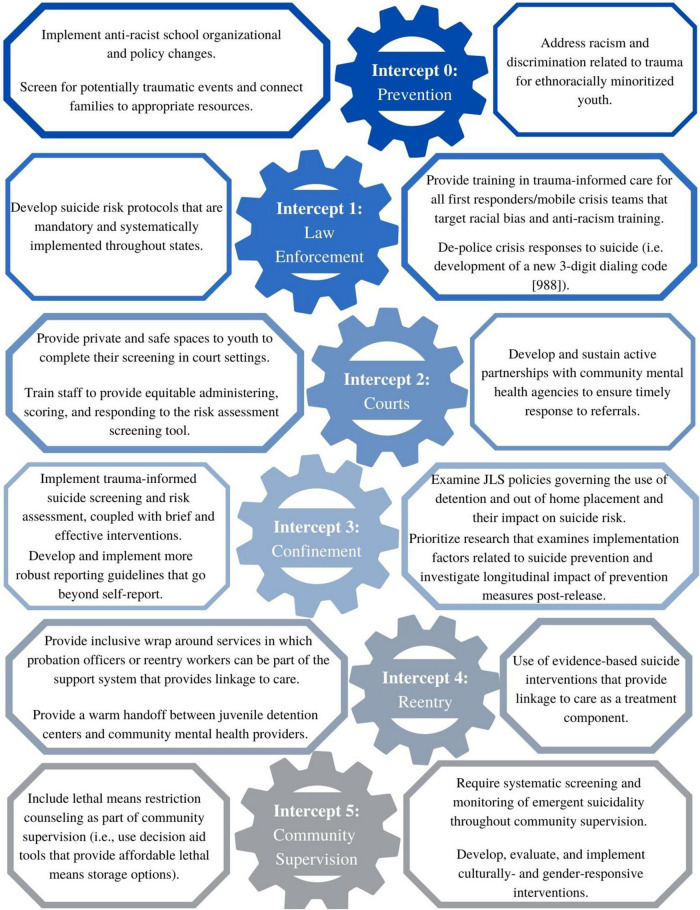
Equitable suicide prevention strategies across the Sequential Intercept Model.

We illustrate disparities/inequities and opportunities for equitable suicide prevention across the SIM for youth impacted by the JLS (see Figure 1) using a Structural Racism and Suicide Prevention Systems Framework (9), which posits that youth are embedded across multiple ecological systems that illustrate how individual, interpersonal, community, and societal factors intercept and influence each other over time and have an impact on suicide risk. Additionally, given that childhood trauma is a significant risk factor for suicide attempts (10) and that ethnoracially minoritized youth are more likely to experience potentially traumatic events (11, 12), we will pay particular attention to efforts disrupting the pathway from trauma to suicidal risk. Within these frameworks, we contend that inequities are compounded across the SIM, and to truly address inequities through culturally and trauma responsive care, we must evolve from individual to structural targets in suicide prevention efforts (9).

### Intercept 0: Prevention

Intercept 0 encompasses prevention of JLS involvement, which in turn, can decrease suicide risk. Equitable prevention should disrupt community and system level pipelines, e.g., school and child welfare, that target and propel ethnoracially minoritized youth into the JLS. School has been a child serving system that can impact both legal system involvement and suicidality. For example, school resource officer programs are related to increased arrests for non-serious violent crimes (e.g., truancy and curfew violations), and higher, disproportionate rates of suspensions and expulsions specifically for ethnoracially minoritized youth (13, 14). Such disciplinary action can lead to school disengagement, a precursor to JLS involvement (15). Additionally, school suspensions/expulsions limit youth from receiving school-based mental health prevention services that can decrease risk factors for suicidality. If youth do receive services, they are frequently related to disruptive behaviors seen outside the context of trauma-related issues, depression, or anxiety (16, 17). School-based mental health services often include universal, selective, and indicated prevention interventions for suicide. Universal prevention programs focus on reducing stigma about suicide and mental health and increase student help-seeking behavior (18). The most common form of suicide prevention in schools, selective prevention, trains peers, teachers, and school staff to identify and intervene in a suicidal crisis (19). Finally, indicated prevention programs target high-risk students focused on reducing suicidal thoughts and behaviors (20). However, if students of color are consistently absent from school, it decreases the likelihood that they will be identified and receive these services. The combination of disproportionate discipline and prevention of mental health service access in schools fails ethnoracially minoritized youth in that it creates a direct pipeline to JLS involvement and reduces their likelihood to access suicide prevention services.

To address the challenges in disrupting these system-level pipelines to JLS involvement, a trauma-informed and trauma-responsive care model has been proposed. This addresses macro level change, e.g., anti-racist school organizational and policy changes, and practice related changes, e.g., screening for potentially traumatic events and connecting families to appropriate resources (17). This culturally appropriate care should also address racism and discrimination related to trauma for ethnoracially minoritized youth (21). Yet, more research is needed on how to disrupt child serving system pipelines through partnering with key stakeholders in the community and working across systems to adopt a unified framework for working with ethnoracially minoritized youth.

### Intercept 1: Law enforcement

Intercept 1 involves diversion performed by law enforcement and other emergency service providers to treatment in the community instead of being arrested or detained. Such diversion is urgently needed, given that youth with high-risk for suicide are more likely to have legal stressors. In a study of youth that died by suicide, an alarming 63% had a JLS referral and 80% of those under 18 had previous JLS contact (22). Police officers have significant potential “therapeutic contact,” given their high contact with youth that are at high risk for suicide, with recent surveys indicating that 84% of police officers encounter an individual with suicide risk as part of their job (23). This suggests that police officers are not only first-responders but also are gatekeepers/crisis liaisons that can divert youth at risk for suicide from the JLS to evidence-based care in the community.

Unfortunately, police officers often do not receive adequate training for detection of youth suicide risk, with data showing that approximately one in four police officers have not received any training in the management or intervention of suicide (23). Some states still do not require the training in suicide protocols. Despite very few studies documenting this phenomenon, the criminalization of suicide by police officers (i.e., perceiving suicidal behaviors as aggressive) is a significant barrier for adequate links to suicide prevention services (24). In order to address these inequities, future research should prioritize the examination of potential biases in policing associated with Black and Latinx suicidal youth.

To address the challenges within this intercept, we recommend compulsory trauma-informed training for all first responders/mobile crises teams that target racial bias. Concerted efforts need to be made to reduce officer stress that activates heuristics that can lead to racist policing practices (25). In addition, we recommend the training of police officers in suicide prevention gatekeeper training, as there is a positive relationship between receiving evidence-based training and outcomes in knowledge, attitudes, self-efficacy, and use of intervention behavior by police officers (23). This can be done through the development of protocols that are mandatory and systematically implemented throughout states. Lastly, in efforts to de-police crisis responses to suicide, a new three-digit dialing code [988] (available July 16, 2022) will connect all callers to trained counselors. Taken together, these strategies prevent law enforcement from providing suicide care without proper training and also highlight community responses to crises.

### Intercept 2: Courts

Intercept 2 involves judicial proceedings within the court. Most JLS involvement tends to end here (26), which may explain why the court system has often been overlooked in suicide prevention. Currently, courts are being empirically evaluated as a place to screen, intervene, and refer to appropriate services for suicidality (27–29). Currently, the MAYSI-2, a screening tool validated within the JLS population, is used to assess suicide risk and triggers further suicide assessment from a mental health worker (28), however, there has been concern with racial/ethnic and gender differential item functioning with the Suicide Ideation and Traumatic Experiences subscales (30). While the procedure to screen, conduct a safety plan intervention, and refer to community-based mental health services has been deemed both feasible and acceptable (28), there are several inequities that come with suicide prevention in the court setting.

Courts are not designed as mental health service facilities, where there can be a lack of privacy for youth in completing screening, treatment, and referral services. Being in the court may pose additional stress and impact reliable screening. Timing of screening is important; if the youth is about to enter the courtroom or recently received an unfavorable sentence, their ability to report relevant risk factors, including hopelessness, is compromised. This is especially relevant for ethnoracially minoritized youth given that they are more likely to receive harsher sentences than their White peers (31). Relatedly, training for the mental health providers conducting the screening and safety plan requires appropriate time, resources, and finances (27). Training should include uniform risk categories or ways to determine which youth are referred to which services (i.e., at what risk level are youth referred to immediate hospitalization or outpatient services) to reduce bias from the assessor. The risk-needs-responsivity model (32) emphasizes the importance of matching intervention with risk level.

Universal screening, along with appropriate risk mapping and referral systems, is a priority. To do this, courts should provide private and safe spaces to youth to complete their screening, ensure their staff has appropriate training to provide equitable administering, scoring, and responding to the risk assessment screening tool. Courts should be active partners with community mental health agencies to ensure timely response to referrals and open communication between parties involved in the youth’s case. Such practice efforts should be continued to be empirically validated as a location for risk assessment and intervention with our present charge to ensure equitable implementation.

### Intercept 3: Confinement

Intercept 3 considers the services needed to prevent the worsening of mental wellness of youth while they are detained awaiting disposition or receiving services in long-term rehabilitative facilities. In juvenile detention, suicide risk screening is common practice at admission, though there is variance in implementation (33). After admissions screening, it is unclear what follow up intervention occurs apart from making an environment safe (e.g., removing sheets from a room and increasing staff observation). Few evidence-based interventions exist for incarcerated adolescents (34), though there has been successful implementation of the CSSR-S and Safety Planning Intervention in secure detention facilities (35). Challenges to suicide prevention in juvenile detention include lack of guidelines to specify evidence-based practice use and staffing capacity of qualified individuals in facilities to do suicide risk screening (36).

We recommend researchers examine implementation factors related to suicide prevention and investigate longitudinal impact of prevention measures post-release (36, 37). More robust reporting guidelines that go beyond self-report and limited institutional data can clearly delineate factors that could influence disparities in care (37). We recommend the implementation of trauma-informed suicide screening and risk assessment, coupled with brief and effective interventions like the Safety Planning Intervention or SAFETY (38). These practices can be implemented by clinical staff and task-shifting strategies can be an implementation strategy to increase capacity of detention staff for suicide screening and intervention. A key component of prevention includes parental/caregiver involvement in care and being intentional about caregiver engagement in treatment (e.g., using family visits as a way for integration).

Diversion from detention is the most upstream, impactful approach to suicide prevention. JLS should examine their policies governing the use of detention and out of home placement, as such placements drastically increase the risk of suicidality (39). Standardized instruments to divert youth from detention may help, although there are risks of inequitable implementation through a process called judicial override, where a judge can detain the youth despite the risk assessment recommending diversion. Bias can factor into such overrides. For instance, if a judge weighs school attendance more than other factors, something not favorable for non-White youth (40), the override can create inequitable detainment for non-White youth.

### Intercept 4: Reentry

Intercept 4, supports reentry back into the community after detention/confinement to increase linkages to care and reduce further JLS involvement through use of reentry coordinators, peer support staff, or community partners. Current barriers to equitable suicide prevention in the reentry period include the challenges with sharing information and coordinating aftercare, a task that is further compounded with the complication of pre-trial status (i.e., multiple probation officers and court staff). From our collective clinical experience, we have noted that the stress of community reentry is a precipitator for emotional distress and suicide risk. In fact, studies indicate that 40–70% of youth recidivate within 1 year (and Black male youth with behavioral problems have a risk for shorter recidivism time) and recidivism is a perpetuating barrier to receiving appropriate suicide treatment (41). In a large study of adolescents, being placed on suicide precaution in confinement was associated with increased recidivism (41).

A significant barrier to suicide prevention during this intercept is the involvement of parents into the care of youth as they transition back home. Parental involvement is a major common treatment element of effective suicide interventions for youth, however, many parents/caregivers report feeling “out of the loop,” regarding their youth’s mental health during reentry which results in difficulty in accessing and utilizing mental health care. Another potential barrier to parental/caregiver involvement in care, is that many youth that are incarcerated also have a parent that is incarcerated. Similarly, long wait periods between detention release and initial contact with court or probation officers is associated with decreased motivation for youth to seek care (42). Mental health treatment seeking during reentry is even lower among Black and Latinx youth (43).

To address current barriers and disparities for suicide during reentry, we propose the provision of inclusive wrap around services in which probation officers or reentry workers can be part of the support system that provides linkage to care. We also propose the warm handoff between juvenile detention centers and community mental health providers and the use of evidence-based interventions that provide linkage to care [e.g., SAFETY; (38)]. An important area for future research that needs to be prioritized is the development and evaluation of culturally responsive suicide interventions for youth and families involved in the JLS as they transition back to their community.

### Intercept 5: Community supervision

Intercept 5 aims to support youth with mental health needs to limit community supervision violations and prevent new offenses. Juvenile probation officers are charged with administering rehabilitation risk/needs assessments, connecting youth to resources related to identified needs, and ensuring compliance with the terms of court orders. While cases on supervision have decreased in response to the decrease in juvenile arrests, those who are on probation have higher risk profiles including trauma histories and suicide risk (44). Despite this increased risk, data shows that only 20% of court, and probation staff screen for suicide (45). This gap may be attitudinal (e.g., “that’s not my job”), limited capacity for training, or logistical, related to limited referral networks.

An integrated screening and referral program that maps risk to the appropriate level of service is needed; however, accessibility remains a challenge, with up to 80% of youth with mental health needs going without receiving appropriate mental health care (46). Care access is an even greater barrier for ethnoracially minoritized youth that live in areas far from community clinics and oftentimes lack the financial resources (and time) for transportation. Clinics themselves may lack resources for evidence-based care [e.g., Dialectical Behavior Therapy (DBT)]. Care coordination may be difficult across systems, as there may be interorganizational confusion (47) in terms of who is responsible for what aspects of a youth’s rehabilitation across different service systems. For instance, with youth that are screened but are placed in shelter care, there is a tenfold increased risk in suicide attempts, highlighting problems with care coordination with multiple systems of care (39).

Our recommendations center on systematic screening and monitoring for emergent suicidality throughout community supervision. We also echo (48) who articulate that cultural and gender specific interventions are warranted especially when suicide risk is viewed through a social determinants of health lens. A research and related practice gap includes understanding the needs of gang involved youth on supervision, as these youth may have intensive probation for violence or gun-related charges and are seven times more likely to have suicide attempts than non-violent-offense youth (39). Such supervision should include restricting access to lethal means as also related to suicide risk.

## Discussion and conclusion

Suicide inequities/disparities will continue to widen if we do not disrupt our current approach to suicide prevention that targets individual level factors with approaches addressing structural determinants of health. Structural racism not only has an impact on prevention of JLS involvement, but it also can perpetuate JLS involvement through unnecessarily long community supervision. Our discussion of the SIM as a way to map suicide prevention/intervention resources aligns with the prevention strategies outlined by Centers for Disease Control and Prevention (49), in which they call for multipronged approach to suicide prevention. The outlined approaches include the strengthening of economic supports, strengthening access and delivery of suicide care, creating protective environments, promoting connectedness, teaching coping and problem-solving skills, identifying and supporting people at risk, using trauma-informed and culturally responsive suicide prevention strategies, and lessening harms and prevention of future risk. This approach underscores a structural response to structural inequities for JLS youth and serves to protect youth from suicide to keep them out of hospitals and the JLS.

## Data availability statement

The original contributions presented in this study are included in the article/supplementary material, further inquiries can be directed to the corresponding author.

## Author contributions

All authors participated in the conceptualization, writing, and editing process of this manuscript and contributed to the article and approved the submitted version.
